# Bispecific CS1-BCMA CAR-T cells are clinically active in relapsed or refractory multiple myeloma

**DOI:** 10.1038/s41375-023-02065-x

**Published:** 2023-10-17

**Authors:** Chenggong Li, Jia Xu, Wenjing Luo, Danying Liao, Wei Xie, Qiuzhe Wei, Yinqiang Zhang, Xindi Wang, Zhuolin Wu, Yun Kang, Jin’e Zheng, Wei Xiong, Jun Deng, Yu Hu, Heng Mei

**Affiliations:** 1 Institute of Hematology, Union Hospital, Tongji Medical College, Huazhong University of Science and Technology, Wuhan, 430022 China; 2 Hubei Clinical Medical Center of Cell Therapy for Neoplastic Disease, Wuhan, 430022 China; 3 Center for Stem Cell Research and Application, Union Hospital, Tongji Medical College, Huazhong University of Science and Technology, Wuhan, 430022 China; 4 Wuhan Sian Medical Technology Co., Ltd Wuhan, Wuhan, 430022 China

**Keywords:** Cancer immunotherapy, Myeloma

## Abstract

Multiple myeloma (MM) bears heterogeneous cells that poses a challenge for single-target immunotherapies. Here we constructed bispecific CS1-BCMA CAR-T cells aiming to augment BCMA targeting with CS1. Sixteen patients with relapsed or refractory (RR) MM received CS1-BCMA CAR-T infusion. Six patients (38%) had cytokine release syndrome, which was of grade 1–2 in 31%. No neurological toxicities were observed. The most common severe adverse events were hematological, including leukopenia (100%), neutropenia (94%), lymphopenia (100%) and thrombocytopenia (31%). Three patients with solitary extramedullary disease (sEMD) did not respond. At a median follow-up of 246 days, 13 patients (81%) had an overall response and attained minimal residual disease-negativity, and six (38%) reached a stringent complete response (sCR). Among the 13 responders, 1-year overall survival and progression-free survival were 72.73% and 56.26%, respectively. Four patients maintained sCR with a median duration of 17 months. Four patients experienced BCMA+ and CS1+ relapse or progression. One patient responded after anti-BCMA CAR-T treatment failure. Lenalidomide maintenance after CAR-T infusion and the resistance mechanism of sEMD were preliminarily explored in three patients. CAR-T cells persisted at a median of 406 days. Soluble BCMA could serve as an ideal biomarker for efficacy monitoring. CS1-BCMA CAR-T cells were clinically active with good safety profiles in patients with RRMM. Clinical trial registration: This study was registered on ClinicalTrials.gov, number NCT04662099.

## Introduction

Bispecific CAR-T cell therapy has been proposed to mitigate some limitations of single-target CAR-T cells. CS1, also known as CD319, SLAMF7, and CRACC, is a cell surface glycoprotein, which is highly expressed on multiple myeloma (MM) cells both at diagnosis and at relapse [1]. CS1-targeted elotuzumab has been approved in treatment of relapsed or refractory multiple myeloma (RRMM) [2, 3]. Irreversible BCMA loss was reported in a few relapsed patients after BCMA-targeted CAR-T cell infusion, but their MM cells maintained CS1 expression [4]. Therefore, we aimed to augment BCMA targeting with CS1 in treatment of MM.

CS1-targeted CAR-T or CAR-NK cells conferred potent and consistent anti-myeloma activity in vitro and in vivo [5–8]. However, CS1 is expressed on a fraction of normal lymphocytes, including NK, T, and B cells [1]. Single CS1-targeted CAR-T cells exhibited selective fratricide of CS1^+/high^ normal lymphocytes but spared CS1^–/low^ fraction and preserved functional lymphocytes [7, 8]. A compound CAR-T cell possessing two complete and independent CARs targeting BCMA and CS1 exerted superior anti-myeloma effects in MM mice models compared to a single-target CAR-T cell [9]. Tandem BCMA/CS1 CAR-T cells maintained superior proliferative capability with minimal fratricide compared to compound CAR-T cells [10]. Prompted by this, we designed a tandem bispecific CS1-BCMA CAR containing a novel anti-CS1 single-chain fragment variable (scFv, clone 7A8D5) [11] and a novel anti-BCMA scFv (clone 4C8A) [12]. Bispecific CS1-BCMA CAR-T cells were effective in targeting MM cells in the preclinical studies [11]. Here we reported the preliminary safety, efficacy, and in vivo kinetics of CS1-BCMA CAR-T cells in patients with RRMM in our phase I clinical trial (NCT04662099). Lenalidomide enhanced the anti-myeloma activity and persistence of CS1-targeted CAR-T cells in MM xenograft models [13]. Hence, we further pioneered lenalidomide maintenance after CS1-BCMA CAR-T cell infusion.

BCMA can be directly cleaved by γ-secretase from MM cell-surface to form soluble BCMA (sBCMA) [14]. sBCMA correlated positively with tumor burden in bone marrow (BM) and disease status, but negatively with survival [15]. Meanwhile, the baseline levels and early changes of sBCMA could predict the clinical responses and prognosis of MM patients [16, 17]. sBCMA in peripheral blood (PB) declined more quickly in the responders after anti-BCMA CAR-T cell treatment compared with nonresponders [18]. CS1 can also be shed by unknown mechanisms to form soluble CS1 (sCS1). sCS1 promoted the growth of MM cells via homophilic interaction with surface CS1 and subsequent activation of the SHP-2 and ERK signaling pathways [19]. Baseline sCS1 was a predictive biomarker for elotuzumab therapy [20]. We longitudinally monitored the changes of sBCMA and sCS1 in PB and BM of treated patients to investigate their predictive value during CS1-BCMA CAR-T cell therapy.

## Methods

### Trial design

A single-arm phase 1/2a clinical trial (NCT04662099) was designed to evaluate the feasibility, safety, and efficacy of bispecific CS1-BCMA CAR-T cells in patients with RRMM. This study was approved by the Ethics Committee of the Union Hospital affiliated to Huazhong University of Science and Technology, Wuhan, China. The study was done in accordance with the principles of the Declaration of Helsinki. All patients provided written informed consents.

The enrolled patients must have received at least two prior lines of therapy, and previous BCMA- or CS1-targeted immunotherapies were allowed. Patients were subjected to lymphodepleting regimens with cyclophosphamide (250 mg/m^2^, d-5 to d-3) and fludarabine (30 mg/m^2^, d-5 to d-3) daily prior to the CAR-T infusion. Planned dose levels were 0.75 × 10^6^, 1.5 × 10^6^, and 3.0 × 10^6^ CAR + T cells/kg, and repeated infusions were allowed. Primary objectives were incidence of adverse events (AEs). Cytokine release syndrome (CRS) and immune effector cell-associated neurotoxicity syndrome (ICANS) were graded using the ASTCT criteria [21], and other AEs using Common Terminology Criteria of Adverse Events version 5.0. Secondary objectives were overall response rate (ORR), overall survival (OS), duration of response (DOR), and progress-free survival (PFS). Response was assessed per the IMWG criteria 2016 [22]. Other objectives included in vivo kinetics of CAR-T cells, the phenotypes of infused CAR-T cells, and the changes of sBCMA and sCS1 in PB and BM.

### CAR construct and CAR-T cell manufacturing

**Fig. 1:**
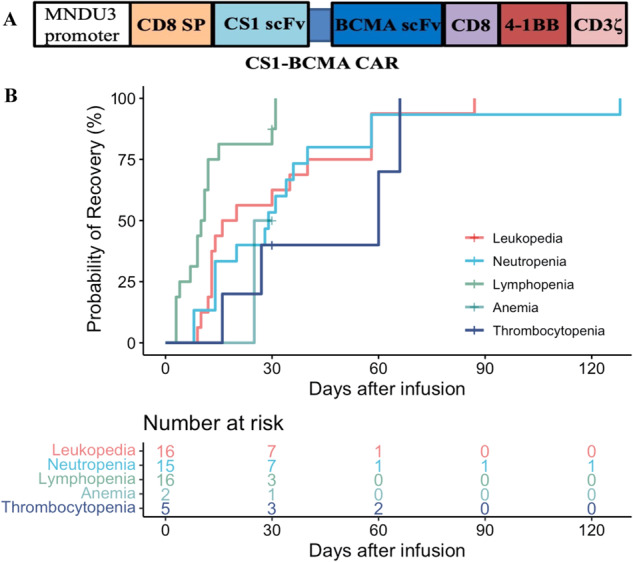
Schematic structure of CS1-BCMA CAR and time to recovery of grade 3-4 cytopenias. **A** Schematic structure of CS1-BCMA CAR containing MNDU3 promoter, CD8 signal peptide (SP), anti-CS1 single-chain fragment variable (scFv) linked with a novel anti-BCMA scFv, CD8 hinge and transmembrane domain, 4-1BB costimulatory domain and CD3ζ signaling domain. **B** Time to recovery of grade 3–4 cytopenias. Patients with grade 3–4 cytopenias (leukopenia (*n* = 16), neutropenia (*n* = 15), lymphopenia (*n* = 16), anemia (*n* = 2) and thrombocytopenia (*n* = 5)) after infusion are included. Recovery is defined as cytopenias of grade 2 or better (absolute leukocyte counts ≥2000 cells/μL, absolute neutrophil counts ≥1000 cells/μL, absolute lymphocyte counts ≥500 cells/μL, hemoglobin concentration ≥80 g/L, or platelets ≥50,000/μL). Time to recovery is defined as the time from infusion to the first time when recovery criteria was met.

Bispecific CS1-BCMA CAR contained a murine anti-CS1 scFv (clone 7A8D5) and a murine anti-BCMA scFv (clone 4C8) in tandem, and a 4-1BB costimulatory domain (Fig. 1A). The detailed information and manufacturing method of CS1-BCMA CAR-T cells were previously described [11, 12]. Briefly, at least 1.2 × 10^9^ blood mononuclear cells were obtained from the enrolled patients by leukapheresis (COBE Spectra, USA). T cells were purified and transfected with lentivirus containing CS1-BCMA CAR sequence and expanded in vitro. Quality control was as previously given [23, 24].

### Laboratory assays

CAR expression was detected using biotinylated human BCMA (BC7-H82FO; ACRO Biosystems) and then stained with streptavidin-PE (405203; Biolegend). BCMA and CS1 expression on MM cells were determined by BCMA-PE (357504, Biolegend) and CS1-APC (331810; Biolegend). CAR-T cell quantitation in PB and BM was determined by flow cytometry (FCM) with precision count beads (424902; Biolegend) as well as by droplet digital polymerase chain reaction (ddPCR). The forward primer was AGAGGAAGATGGCTGTAG; the reverse primer was CTGCTGAACTTCACTCTC; the probe sequence was FAM-CACATCCTCCTTCTTCTTCTTCTGG-TAMRA (Tsinke, China). The levels of sBCMA and sCS1 in PB and BM was quantified by enzyme-linked immunosorbent assay (Human BCMA/TNFRSF17 DuoSet ELISA, VAL130, NOVUS; Human SLAMF7 ELISA Kit, OKCD00598, Aviva). The phenotypic characteristics of infused CAR-T cells was systematically evaluated by multicolor FCM using ID7000 full-spectrum flow cytometry (Sony). The antibodies details for FCM, immunohistochemistry, and immunofluorescence were provided in Table S1. RNA sequencing was finished on an Illumina Nextseq 500 platform. The raw data were uploaded to SRA database and the Accession ID was PRJNA1023033. Other relevant evaluation was detailed as previously [23].

### Statistical analysis

Descriptive statistics include means with 95% confidence intervals (CI) or median with range for continuous variables and counts with percentages for categorical variables. Missing data were not imputed. Fisher’s exact test was used for categorical variables. Continuous variables were compared using t test or one-way ANOVA. DOR, PFS, and OS of patients, and in vivo survival of CAR-T cells were determined by using the Kaplan–Meier methods and compared by utilizing the log-rank test. Pearson correlation analysis was used. All analyses were performed with GraphPad Prism 7. *P* < 0.05 (two-tailed) was considered significant.

## Results

### Patient characteristics

As of October 30, 2022, 18 patients were enrolled, and 16 patients had received CS1-BCMA CAR-T cell infusion. Two patients died of disease progression during CAR-T cell manufacturing (Fig. S1). The baseline characteristics of the treated patients are shown in Table 1. Because of osteolytic damage caused by MM, 50% of the patients had an ECOG score greater than one. Nine patients (56%) carried high-risk cytogenetic abnormalities, and 69% had stage III disease according to R-ISS staging. Six patients (38%) had extramedullary disease (EMD) and three patients (19%) had solitary EMD (sEMD) without detectable MM cells in their BM. The median MM blasts in BM defined by immunophenotyping were 5.93% (range, 0–70). The median BCMA and CS1 expression on MM cells was 81.12% (range, 38.88–98.36) and 98.64% (range, 91.58–99.14), respectively. The median prior lines of treatment were 4 (range, 2–8). The detailed previous therapeutic regimes are shown in Table S2. Seven patients (44%) received autologous stem cell transplantation (ASCT), five (31%) were exposed to daratumumab due to an access issue, two (13%) received selinexor and two (13%) had previous BCMA-targeted CAR-T cell therapy. All patients (100%) were exposed to bortezomib, six (38%) to ixazomib and one (6%) to cafilzomib. Fifteen patients (94%) received lenalidomide, eight (50%) received thalidomide and four (25%) received pomalidomide.

**Table 1:** Baseline Characteristics of the Patients.

All evaluable patients, *n* = 16	Median (range) or No.(%)
Age (years)	60 (42–73)
Gender	
male	8 (50)
female	8 (50)
ECOG performance status	
1	8 (50)
2	3 (19)
3	4 (25)
4	1 (6)
Monoclonal type	
IgG	7 (44)
IgA	4 (25)
IgM	1 (6)
k light chain	3 (19)
λ light chain	1 (6)
EMD	
with EMD	6 (38)
solitary EMD	3 (19)
Complex karyotype	1 (6)
Cytogenetic profiles^a^	9 (56)
R-ISS stage	
I	3 (19)
II	2 (13)
III	11 (69)
Time since diagnosis, months	42 (5–96)
MM burden at enrollment	
Serum M protein (g/L)	4.1 (0–63.6)
MM cells in BM by morphology (%)	11 (0–89)
MM cells in BM by FCM (%)	5.93 (0–70)
BCMA + MM (%)	81.12 (38.88–98.36)
CS1 + MM (%)	98.64 (91.58–99.14)
Prior lines of therapies	4 (2–8)
Prior ASCT	7 (44)
Prior anti-BCMA CAR-T therapy	2 (13)
Prior daratumumab	5 (31)
Prior Selinexor	2 (13)
Prior proteasome inhibitors	
Bortezomib	16 (100)
Carfilzomib	1 (6)
Ixazomib	6 (38)
Prior immunomodulatory drugs	
Lenalidomide	15 (94)
Thalidomide	8 (50)
Pomalidomide	4 (25)

*BM* bone marrow, *MM* mutiple myeloma, *ECOG* eastern cooperative oncology group, *FCM* flow cytometry, *ASCT* autologous stem cell transplantation, *EMD* extramedullary plasmacytoma.

^a^Cytogenetic profiles include amp(1q21), del(17p), t(4;14), t(14;16), and t(11;14).

### Safety

All the patients had AEs of grade 3 or higher (Table 2). Six patients (38%) had CRS, with a median onset of 8 (range, 6–12) days and a median duration of 4 (range, 3–7) days. Grade 3 CRS occurred once and lasted for 5 days. Glucocorticoids were given to the six patients and two received tocilizumab. ICANS was not observed. Headache and limb numbness were observed in two patients, respectively. Infection occurred in six patients (38%), and grade 3 infection in five patients (31%). Despite the subgroup analysis limited to the small sample size, the occurrence of CRS was associated with the baseline levels of β microglobulin, BCMA+ ratio in MM cells, the peak levels of serum interferon-γ and ferritin, and the ratio of peak to baseline of serum IL-6, interferon-γ and ferritin (Table S3).

**Table 2:** Adverse Events within 2 Months after Infusion.

Adverse events	Any Grade	Grade1	Grade2	Grade 3	Grade 4
	No.(%)				
CRS	6 (38)	3 (19)	2 (13)	1 (6)	0
ICANS	0	0	0	0	0
Fever	9 (56)	4 (25)	4 (25)	1 (6)	0
Headache	2 (13)	0	2 (13)	0	0
Pain	6 (38)	1 (6)	2 (13)	3 (19)	0
Fatigue	4 (25)	4 (25)	0	0	0
Limb numbness	2 (13)	2 (13)	0	0	0
Edema of both lower limbs	6 (38)	5 (31)	0	1 (6)	0
Cough	4 (25)	2 (13)	2 (13)	0	0
Infection	6 (38)	0	1 (6)	5 (31)	0
Gastrointestinal symptoms^a^	5 (31)	3 (19)	1 (6)	1 (6)	0
Leukopenia	16 (100)	0	0	7 (44)	9 (56)
Neutropenia	16 (100)	0	1 (6)	6 (38)	9 (56)
Lymphopenia	16 (100)	0	0	0	16 (100)
Anemia	4 (25)	0	2 (13)	2 (13)	0
Thrombocytopenia	10 (63)	3 (19)	2 (13)	1 (6)	4 (25)
AST increase	4 (25)	2 (13)	2 (13)	0	0
ALT increase	5 (31)	3 (19)	0	1 (6)	1 (6)
Hypofibrinoemia	3 (19)	1 (6)	2 (13)	0	0
Hyponatremia	2 (13)	2 (13)	0	0	0
Hypokalemia	10 (63)	6 (38)	3 (19)	1 (6)	0
Hypocalcemia	4 (25)	4 (25)	0	0	0
Hypomagnesemia	7 (44)	6 (38)	1 (6)	0	0

*CRS* cytokine release syndrome, *ICANS* immune effector cell associated neurotoxicity syndrome, *ALT* alanine aminotransferase, *AST* aspartate aminotransferase.

^a^Gastrointestinal symptoms included grade 1 abdominal distension, grade 1 diarrhea, grade 1 constipation, grade 2 nausea, and grade 3 gastrointestinal bleeding.

Hematological toxicities were the most common AEs; grade 3–4 hematological AEs were leukopenia (in 100% of the patients), neutropenia (in 94%), lymphopenia (in 100%), anemia (in 13%) and thrombocytopenia (in 31%) (Table 2). The median time for patients with grade 3–4 cytopenia that recovered to grade 2 or less were 18 days for leukopenia, 29 days for neutropenia, 10.5 days for lymphopenia, 25 days for anemia, and 60 days for thrombocytopenia (Fig. 1B). Five patients (31%) were observed with hepatotoxicity, manifested by elevated alanine aminotransferase and aspartate aminotransferase, and all recovered within one month after infusion. All patients had preexisting hypoimmunoglobulinaemia that deteriorated after infusion. Patients 2 and 6 were restored to normal serum immunoglobulin levels in 18 months, and other patients remained hypoimmunoglobulinaemia during the follow-up (Fig. S2). Five patients (31%) experienced six infections within 2 months after infusion, including one bacterial conjunctivitis of grade 2 and five pulmonary infections of grade 3. Patient 4 had gastrointestinal bleeding secondary to thrombocytopenia. Platelet infusion, inhibition of gastric acid secretion, and other supportive treatments were effective. All the toxicities of CS1-BCMA CAR-T cell therapy were manageable.

### Efficacy

**Fig. 2:**
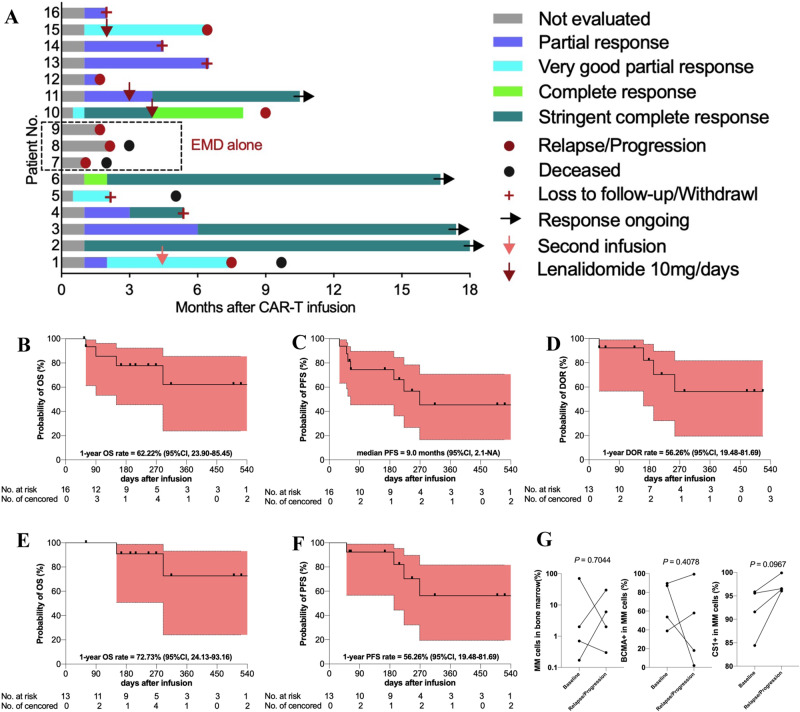
Clinical outcomes of CS1-BCMA CAR-T cell therapy. **A** Swimmer plot for the 16 treated patients. Patients 7, 8 and 9 had solitary EMD without detectable MM cells in bone marrow. Kaplan–Meier curves for overall survival (OS) (**B**), progression-free survival (PFS) (**C**), and duration of response (DOR) (**D**) of the 16 treated patients. Kaplan–Meier curves for OS (**E**) and PFS (**F**) of the 13 responders. Shaded region indicates 95% CI. **G** Changes of MM cell burdens in bone marrow and their BCMA+ and CS1+ ratios from baseline to disease progression or relapse for the four patients (1, 10, 12 and 15). Paired *t* test was used.

The ORR was 81% for all the 16 treated patients, including six (38%) stringent complete responses (sCR), three (19%) very good partial responses (VGPR), and four (25%) partial responses (PR) (Fig. 2A). Three patients (7, 8 and 9) with sEMD did not respond to CS1-BCMA CAR-T cell treatment. The ORR and sCR rate were 100% and 46% for the 13 patients with detectable MM cells in BM, respectively. The median time to the first PR or better was 1.0 (range, 0.5–1.0) month. Responses deepened over time in some patients, and the median time to the best response was 1.9 (range, 1.0–6.0) months. Patients 7 and 15 had prior anti-BCMA CAR-T cell infusion, patient 7 with sEMD did not respond and patient 15 got VGPR in month 1. All the 13 patients with detectable MM cells in BM (100%) achieved minimal residual disease (MRD)-negativity (≤10^−5^ nucleated cells). The median time to the first MRD-negativity was 0.5 (range, 0.5–1.0) month.

Despite the subgroup analysis limited to the small sample size, the remission depth of the 13 responders was independent with the baseline MM burden, BCMA+ and CS1+ ratio of MM cells, the baseline levels of sBCMA in PB and BM, serum M protein, albumin, β macroglobulin, and LDH, R-ISS staging, the treatment history of ASCT, CAR-T cells, and daratumumab, but correlated to the baseline levels of serum IL-6 and IL-10 (Table S4).

Second infusion was given to patient 1 and oral lenalidomide maintenance (10 mg/day, 21 days of 28-day cycle) was administrated to patients 10, 11 and 15. Patient 1 achieved VGPR in month 3 and received a second infusion in month 4 for reaching a deeper remission. However, patient 1 relapsed in month 8 and withdrew from the study for salvage therapy. Patient 10 was given with lenalidomide in month 4 due to slightly elevated serum free chain, but his disease progressed in month 9. Patient 11 achieved PR in month 1, received lenalidomide in month 3 and reached sCR in month 4. Patient 15 achieved VGPR in month 1 and received lenalidomide in month 2, but his disease progressed in month 6.

At a median follow-up of 246 (range, 55–547) days, the median OS was not reached (NR), and 1-year OS was 62.22% (95%CI, 23.90–85.45) (Fig. 2B). The median PFS was 9.0 (95%CI, 2.1–NR) months (Fig. 2C). The median DOR was NR, and 1-year DOR was 56.26% (95%CI, 19.48–81.69) (Fig. 2D). Four patients (1, 5, 7 and 8) died of disease progression. Among the 13 responders, the median OS and PFS were NR, and 1-year OS and PFS were 72.73% (95%CI, 24.13–93.16) and 56.26% (95%CI, 19.48–81.69), respectively (Fig. 2E, F). Four patients (2, 3, 6 and 11) maintained sCR with a median duration of 17 (range, 10.5–18.2) months. Five patients (4, 5, 13, 14 and 16) were lost due to poor compliance, long medical distance, and COVID-19. Four patients (1, 10, 12 and 15) experienced BCMA+ and CS1+ disease progression or relapse during the follow-up (Fig. 2G).

### Preliminary mechanism exploration for nonresponse to sEMD

**Fig. 3:**
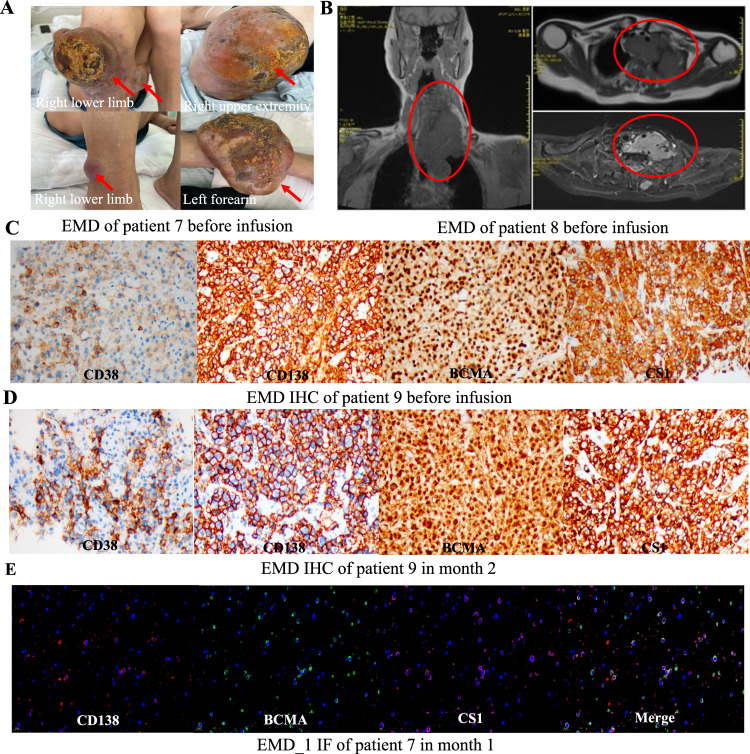
Solitary extramedullary diseases (sEMD) and their BCMA and CS1 expression. **A** Pictures of extensive EMDs of patient 7 before infusion. **B** Computer tomography scans of bulk EMD of patient 8 before infusion. **C** Immunohistochemistry (IHC) for CD38, CD138, BCMA and CS1 of EMD of patient 9 before infusion. **D** IHC for CD38, CD138, BCMA and CS1 of EMD of patient 9 in month 2 after CS1-BCMA CAR-T cell infusion. **E** Immunofluorescence (IF) for CD138, BCMA and CS1 of the original lesion EMD_1 of patient 1 in month 1 after infusion.

Patient 7 had extensive EMDs involving the upper and lower limbs (Fig. 3A). The EMD (82.98 mm × 68.70 mm × 65.56 mm) of patient 8 existed in the left cervical root-upper mediastinum, lung apex, and thoracic vertebrae, and partial fusion compressed the left lung apex, trachea, and esophagus (Fig. 3B). Patient 9 had the EMD (2 cm × 2 cm) with >80% of CD138 + BCMA + CS1+ plasma cells at the 10th rib of the left posterior axillary (Fig. 3C). Unfortunately, the EMD of patient 8 did not shrink, and patients 7 and 9 had newly-onset EMD after infusion. Their EMDs maintained BCMA and CS1 expression in month 1 (Fig. 3D, E).

**Fig. 4:**
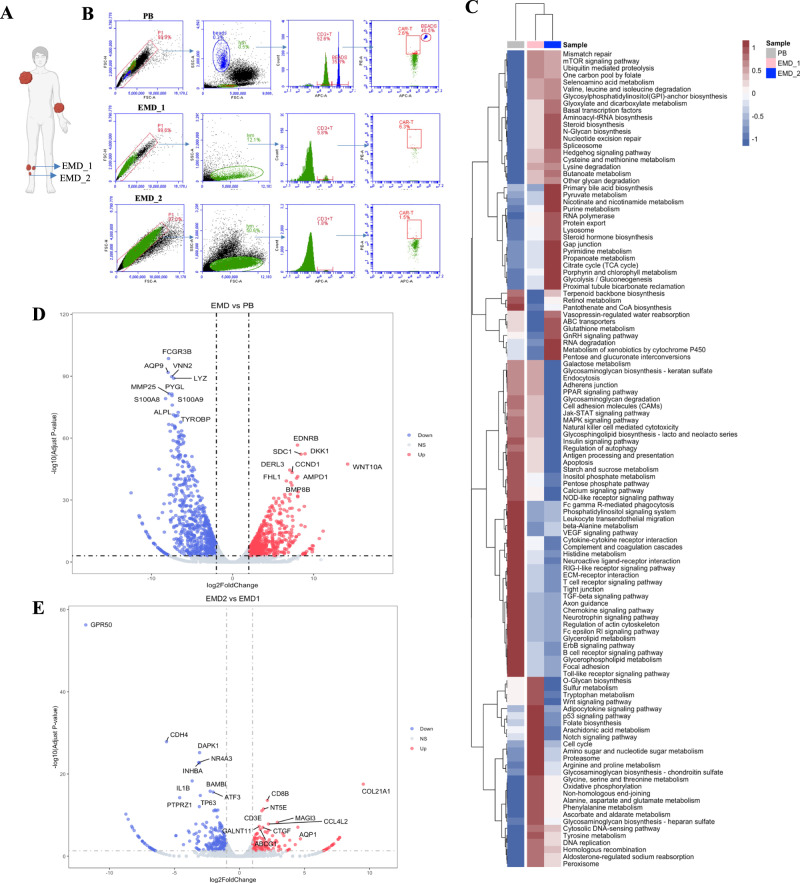
CAR-T cell infiltration and transcriptional analysis of solitary extramedullary diseases (sEMD). **A** Schematic diagram of the EMDs of patient 7 in month 1 after infusion. **B** T and CAR-T cell analysis of peripheral blood (PB), EMD_1 and EMD_2 of patient 7 by flow cytometry. Cells were gated by forward and side scatter, then singlets (P1), then lymphocytes and/or beads for absolute counting, and then CD3 + T and CAR + CD3 + T cells. **C** Gene set variation analysis of PB, EMD_1 and EMD_2 of patient 7 in month 1. **D** Volcano map of differential genes of EMD compared to PB, with marked 10 differentially upregulated genes and 10 differentially downregulated genes. **E** Volcano map of differential genes of EMD_2 compared to EMD_1, with marked 10 differentially upregulated genes and 10 differentially downregulated genes.

To investigate the potential causes of therapeutic resistance of sEMD, we chose the PB sample, the original lesion EMD_1 and the newly-onset lesion EMD_2 of patient 7 in month 1 after infusion for CAR-T cell detection and transcriptome sequencing (Fig. 4A). Although the low-level circulating CAR-T cells (7.13 CAR + T cells/ul) existed in the PB, only 5.8% of tumor infiltrating T cells and 0.37% of CAR + T cells were detected in EMD_1, and 1.8% of T cells and 0.03% of CAR + T cells were detected in EMD_2 (Fig. 4B). Gene set variation analysis found that compared with the PB samples, the differential gene sets of EMD were mainly enriched in metabolic and biosynthetic pathways. The upregulated gene sets mainly included mismatch repair and nucleotide splicing repair, basic transcription factors and splicers, mTOR signaling pathway, Hedgehog signaling pathway, gap junction and other signaling pathways. The downregulated gene sets mainly included cell adhesion molecules, Jak-STAT signaling pathway, MAPK signaling pathway, autophagy regulation, apoptosis, cytokine-cytokine receptor interaction, and TGF-β signal pathway (Fig. 4C). Compared with the PB, 1249 differentially upregulated genes, including EDNRB, DKK1, SDC1, WNT10A, and DERL3, and 1126 differentially downregulated genes, including FCGR3B, AQP9, VNN2, LYZ, and MMP25, were identified in the EMD samples (Fig. 4D). Compared with the EMD_1, 127 differentially upregulated genes and 210 differentially downregulated genes, were identified in EMD_2 (Fig. 4E). The significantly upregulated gene (log2(FC) > 5) was COL21A1, and the significantly downregulated gene (log2(FC) < −5) was CDH4.

### In vivo kinetics

CS1-BCMA CAR + T cells accounted for 55.27% (range, 34.90–78.00) of the final infused cell products, with 58.45% (range, 23.30–88.50) of CD4^+^CAR^+^T cells and 38.05% (range, 10.50–71.10) of CD8^+^CAR^+^T cells (Table S5).

FCM and ddPCR were used to monitor CAR-T cell kinetics in PB and BM. Paired analysis showed a high correlation in the quantification of CAR-T cells in PB and BM (ddPCR: *r* = 0.7236, *P* < 0.001; FCM: *r* = 0.8214, *P* < 0.001) (Fig. S3A, B). FCM and ddPCR showed a correlation in the quantification of CAR-T cells with higher consistency in PB (PB: *r* = 0.7452, *P* < 0.001; BM: *r* = 0.4862, *P* < 0.001) (Fig. S3C, D). Therefore, CAR-T cell kinetics in PB were used for further analysis.

**Fig. 5:**
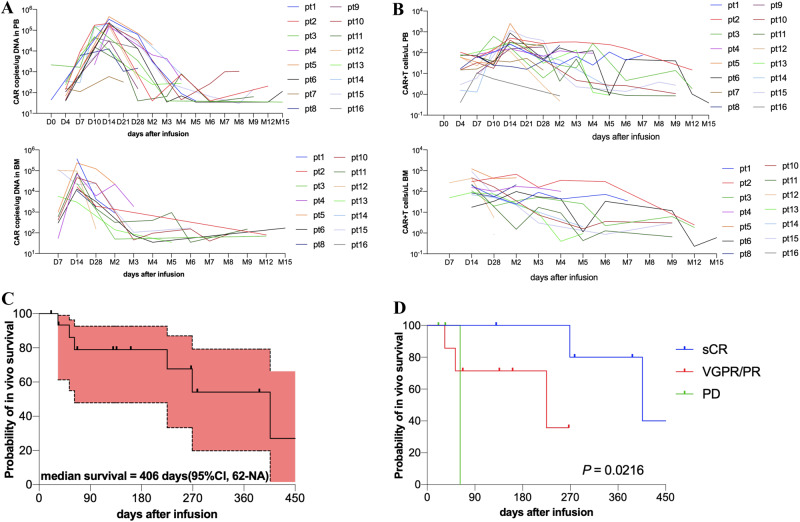
In vivo expansion and persistence of CS1-BCMA CAR-T cells. **A** CAR-T cell kinetics was measured as copies/ug of genomic DNA by digital droplet PCR in peripheral blood (PB) (*n* = 16) and in bone marrow (BM) (*n* = 14). **B** CAR-T cell kinetics was measured as CAR + T cells/ul PB (*n* = 16) and BM (*n* = 14) by flow cytometry. **C** Kaplan–Meier curve of in vivo survival of CS1-BCMA CAR-T cells. Time of survival is defined as the time from infusion to the first time when CAR copies could not be detected by digital droplet PCR. **D** Kaplan–Meier curves of in vivo survival of CAR-T cells in patients with stringent complete response (sCR), very good partial response (VGPR)/partial response (PR), and progressive disease (PD). Log-rank test was used for subgroup comparison.

CS1-BCMA CAR-T cells peaked at 154,250 (range, 590–454,500) copies/ug DNA in PB (*n* = 16) on a median of 14 (range, 10–14) days after infusion by ddPCR. The expansion curve in the first 28 days after infusion (AUC_0-28_) was 252 958 (range, 1370–1,069,170) copies/ug DNA×day (Fig. 5A). By FCM, CAR-T cells peaked at 286.2 (range, 10.7–2554) CAR + T cells/ul PB (*n* = 16) on a median of 14 (range, 7–30) days after infusion. AUC_0-28_ was 643.6 (range, 13.7–2970) CAR + T cells/ul×day (Fig. 5B). Peak expansion and AUC_0-28_ were not associated with the infused dose, clinical response, and the occurrence of CRS (Fig. S4). CAR was detectable in vivo at a median of 406 (range, 62–NR) days (Fig. 5C). Longer in vivo persistence of CAR-T cells was observed in patients who reached deeper remission (Fig. 5D).

### Phenotypic analysis of infused CAR-T cells

CAR^+^ T cells had higher proportion of CD4^+^T, terminal differentiation T (45RA^+^62 L^-^), CCR3^+^CD4^+^T, regulatory T cells (Tregs), PD1^+^T, TIM3^+^T cells, lower proportion of CD8^+^T, and effector memory T (45RA^-^62L^-^) cells compared with CAR^-^ T cells (Table S6). These results indicated that CS1-BCMA CAR transduction was inclined to CD4^+^T cells and further promoted their differentiation and exhaustion. CAR^+^T cells retained as similar CS1 expression as CAR^-^T cells. Subgroup analysis demonstrated that the infused CAR^+^T cells had higher proportions of CD4^+^T and TIM3^+^T cells in the responders. The patients with sCR had higher proportion of TIM3^+^CAR^+^T cells than those with VGPR or PR (Table S7). The patients with CRS had higher ratio of CAR^-^ Tregs than those without CRS (Table S8).

### sBCMA and sCS1

**Fig. 6:**
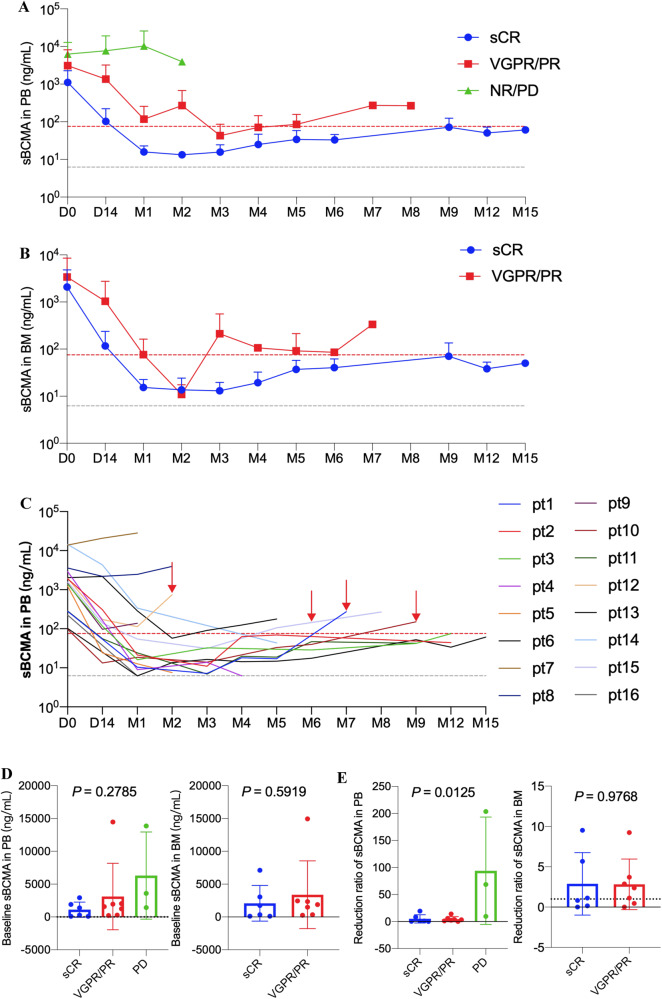
Longitudinal changes of sBCMA in patients treated with CS1-BCMA CAR-T cells. **A** Longitudinal change of sBCMA in peripheral blood (PB) of patients with stringent complete response (sCR), very good partial response (VGPR)/partial response (PR), and nonresponse (NR)/ progressive disease (PD). **B** Longitudinal changes of sBCMA in bone marrow (BM) of patients with sCR and VGPR/PR. Three patients with solitary extramedullary diseases did not respond and their BM samples were not accessible. **C** Longitudinal changes of sBCMA in peripheral blood (PB) of individual patients. Gray dashed line indicated the lower detection limit of sBCMA (6.25 ng/mL) and red dashed line indicated the upper limit of normal value (75.35 ng/mL). **D**, **E** Baseline levels and the reduction ratio of sBCMA in month 1 relative to the baseline in PB and BM of patients with different clinical response. One-way ANOVA was used for PB samples and unpaired *t* test was used for BM samples. Pt patient, M month.

Longitudinal evaluation of sBCMA in PB and BM revealed a rapid decline in the responders after CS1-BCMA CAR-T cell infusion. sBCMA dropped and maintained within the normal levels in patients with sCR but increased slowly beyond the normal levels in patients with VGPR and PR (Fig. 6A, B). The four patients with definite recurrence or progression showed synchronous or advanced increasement in sBCMA beyond upper limit of normal value (Fig. 6C). The clinical response was independent with the baseline levels of sBCMA but correlated to the reduction ratio of sBCMA in month 1 relative to the baseline (Fig. 6D, E). The PB and BM samples from 14 patients were obtained to monitor the sCS1 levels. But the sCS1 levels were not detectable in over 90% of samples and further analysis was not performed (Table S9).

## Discussion

Heterogeneous MM cells present a major challenge for single-target immunotherapies [25–27]. Bispecific CAR-T cells have the theoretical advantages to target broader MM cell populations and mitigate single-target escape. BCMA and CS1 are highly expressed on MM cells [1, 28], regarded as promising targets of immunotherapies for MM. In our trial, 13 patients had MM cell infiltration in their BM with 81.12% of BCMA expression and 98.64% of CS1 positivity. BCMA loss was observed in a few relapsed patients after anti-BCMA CAR-T cell therapy, but their MM cells maintained CS1 expression [4]. Hence, we constructed CS1-BCMA CAR-T cells aiming to augment BCMA targeting with CS1. CS1-BCMA CAR-T cells induced an ORR of 100%, a sCR rate of 46%, MRD-negativity of 100%, 1-year OS and PFS of 72.73% and 56.26%, respectively, in the 13 treated patients with MM cells in their BM. CS1-BCMA CAR-T cell therapy also showed good safety profiles with CRS of 38% and without ICANS.

A concern associated with CS1-targeted CAR-T cells was their selective fratricide of CS1^+/high^ normal lymphocytes, which potentially led to profound lymphopenia and infectious complications [7, 8]. In CARTITUDE-1 trial (*n* = 97), grade 3–4 lymphopenia occurred in 99.0% of patients and 87.5% recovered to grade ≤2 with 1 month [29]. Infections occurred in 58% of patients and 10.3% were grade 3–4 pneumonia [30]. In CARTIFAN-1 trial (*n* = 48), grade 3–4 lymphopenia occurred in 91.7% of patients and grade 3–4 pneumonia was observed in 31.3% [31]. The clinical results of CS1-targeted CAR-T cells are not yet available. CS1-targeted elotuzumab did not increase the risks of lymphopenia and infections in patients with RRMM [3]. In our trial (*n* = 16), grade 3–4 lymphopenia occurred in 100% of patients and 87.5% recovered to grade ≤2 with 1 month. Infections occurred in 38% and 31% were grade 3–4 pneumonia. In the final CS1-BCMA CAR-T cell products, CAR^+^ T cells retained as similar CS1 expression as CAR^-^ T cells. CS1-BCMA CAR-T cells displayed weaker killing to CS1^+^ target cells compared to single CS1-targeted CAR-T cells [10]. CS1-targeting seemingly did not cause on-target off-tumor toxicities in the CS1-BCMA CAR, which might correlate to its structural conformation and requires further studies.

Despite subgroup analysis limited to the small sample size, the clinical remission depth was associated with the baseline levels of serum IL-6 and IL-10, the proportion of infused TIM3^+^CAR-T cells, and in vivo survival of CAR-T cells. These are the room for improvement of CS1-BCMA CAR-T cell therapy. Lenalidomide could enhance the anti-myeloma activity and persistence of CS1-targeted CAR-T cells in MM xenograft models [13]. We pioneered oral lenalidomide maintenance (10 mg/day, 21 days of 28-day cycle) in three patients. Patient 10 and 11 reached a deeper response, but patient 10 and 15 finally relapsed or progressed, and patient 11 kept ongoing sCR. Lenalidomide maintenance after CAR-T cell infusion needs further investigation. Encouragingly, the remission depth was independent with MM burden, BCMA+ and CS1+ ratios on MM cells, the levels of sBCMA and serum M protein, R-ISS stage at baseline, and previous treatment including ASCT, CAR-T cells and daratumumab. CS1-BCMA CAR-T cells provide hope for patients who has failed after targeted therapy or had heavy tumor burden.

Solitary plasmacytoma is a rare form of plasma cell dyscrasia, accounting for 2.8–5% [32]. sEMD indicates a significantly worse prognosis [33]. The exact molecular mechanisms underlying extramedullary spread and treatment-resistance are not fully understood. Poor CAR-T cell infiltration is a main reason for nonresponse to sEMD as well as the challenge for solid tumors. Gene set variation analysis identified that mTOR signaling pathway was upregulated and Jak-STAT signaling pathway was downregulated in EMD. mTOR signaling and cellular metabolism are mutual determinants in cancer [34]. JAK/STAT pathway constitutes a rapid membrane-to-nucleus signaling module and induces the expression of various critical mediators of cancer [35]. WNT10A belongs to the Wnt family and interacts with syndecan-1 on the MM cell-surface to mediate their proliferation, migration, resistance, and osteolytic lesions by impairing osteoblast differentiation [36]. To further explore the roles of these signaling pathways and key molecules in sEMD will provide new directions for elucidating the formation and resistance mechanisms of sEMD and provide implications for combined therapy of CAR-T cells.

Under the pressure of CS1-BCMA CAR-T cells, CS1 and BCMA on the MM cell-surface can be cleaved to form sBCMA and sCS1. Therefore, it is vital to monitor the longitudinal changes of sBCMA and sCS1 in patients during CAR-T cell therapy. In our study, the clinical response was independent with sBCMA at baseline, but corelated to its decline rate in month 1. The change tendency of sBCMA could promptly and sensitively reflect the remission status. Per the IMWG criteria [22], the efficacy evaluation of MM is mainly based on serum M protein and free light chains. The monoclonal immunoglobulin had a long metabolism time (21 days for immunoglobulin G) and patients often showed a delayed optimal response [30, 31, 35]. For non-secretory MM, serum M protein and free light chains cannot be detected. sBCMA had a short metabolism time, with a half-life of 36 hours in mice [37]. Moreover, sBCMA could indicate MM burden of patients with non-secretory MM [38]. Collectively, sBCMA can serve as an ideal biomarker for efficacy monitoring and clinical management for MM patients. Due to sCS1 were below the detection limit (0.312 ng/mL) in the majority of samples, further analysis was prohibited. The sCS1 levels ranged from 0.08 to 197 ng/mL, with a median value of 6.9 ng/mL in patients treated with elotuzumab [20]. The sensitivity of sCS1 detection needs to be improved to monitor the low-level sCS1 in MM patients.

Limitations existed in this study. First, the observation was limited to the small number of patients treated and the relatively short follow-up time. The long-term outcomes require further evaluation. Second, the enrolled patients had worse baseline characteristics, including half of patients with an ECOG score greater than 1, three patients with sEMD and two patients who died of disease progression during CAR-T cell manufacturing. Third, five patients were lost for poor compliance, long medical distance, and COVID-19. Strict inclusion criteria and good compliance of patients need to be considered in further studies. Moreover, the treated patients had not been previously exposed to CS1-targeted immunotherapies. Further trails are required to investigate the efficacy of CS1-BCMA CAR-T cells in this population.

Our study firstly demonstrated CS1-BCMA CAR-T cells were clinically active with a good safety profile in patients with RRMM, even after anti-BCMA CAR-T cell therapy. CS1-BCMA CAR-T cells did not induce obvious fratricide and increased the risk of infections. Immune escape caused by target downregulation was not observed. sBCMA could be a predictive biomarker for CS1-BCMA CAR-T cell therapy. Improving the infiltration, persistence, and phenotype of CAR-T cells are critical strategies for clinical investigation to improve long-term prognosis of patients. Developing off-the-shelf CAR-T cells is a promising avenue to provide patients with a more convenient, accessible, and effective therapeutic option.

### Supplementary information

Supplemental materials

## Data Availability

The datasets that support the findings of this study are included in the article. Further inquiries can be directed to the corresponding authors.
