# Supplementary material for: Bispecific CS1-BCMA CAR-T cells are clinically active in relapsed or refractory multiple myeloma
Source: Leukemia. 2023 Oct 17;38(1):149–59. doi: 10.1038/s41375-023-02065-x (PMC10776387; doi:10.1038/s41375-023-02065-x)
Supplement: Supplementary file 1 — Supplemental materials [file 41375_2023_2065_MOESM1_ESM.docx]

**Supplemental materials**

**Table S1. Detailed information of antibodies used in the study**

| **Antibodies** | **Manufacturer** | **Cat Number** |
| --- | --- | --- |
| **Flow cytometry** | | |
| biotinylated human BCMA | ACRO Biosystems | BC7-H82FO |
| PE-streptavidin | Biolegend | 405203 |
| APC anti-human CD3 | Biolegend | 317318 |
| APC anti-human CD3 | BD Biosciences | 561811 |
| FITC anti-human CD4 | Biolegend | 317408 |
| PerCP-Cy5.5 anti-human CD8 | Biolegend | 344708 |
| BV510 anti-human CD45 | BD Biosciences | 662912 |
| PE-Cy7 anti-human CD19 | BD Biosciences | 560728 |
| APC anti-human CD138 | BD Biosciences | 347193 |
| BV421 anti-human CD38 | BD Biosciences | 562444 |
| APC-Cy7 anti-human CD56 | Biolegend | 362512 |
| PE anti-human clambda | BD Biosciences | 555797 |
| FITC anti-human ckappa | BD Biosciences | 555791 |
| FITC anti-human CD56 | BD Biosciences | 562794 |
| PerCP-Cy5.5 anti-human CD45 | BD Biosciences | 564105 |
| APC-Cy7 anti-human CD38 | BD Biosciences | 560676 |
| BV421 anti-human CD138 | BD Biosciences | 562935 |
| APC anti-human CD319 (CS1) | BioLegend | 331810 |
| PE anti-human CD269 (BCMA) | BioLegend | 357504 |
| Zombie UV™ Fixable Viability Kit (DAPI) | Biolegend | 423108 |
| BV510 anti-human CD4 | BD Biosciences | 562970 |
| APC-Cy7 anti-human CD8 | BioLegend | 344713 |
| FITC anti-human CD62L | BD Biosciences | 555543 |
| PerCP-Cy5.5 anti-human CD45RA | BD Biosciences | 563429 |
| PE-Cy7 anti-human CD25 | BioLegend | 356108 |
| BV421 anti-human CD127 | BioLegend | 351310 |
| PerCP-Cy5.5 anti-human CD3 | BioLegend | 300328 |
| FITC anti-human CD4 | BioLegend | 317408 |
| BV421 anti-human CXCR3 | BioLegend | 353716 |
| BV510 anti-human CCR4 | BioLegend | 359416 |
| PE-Cy7 anti-human CD8 | BioLegend | 344712 |
| APC-Cy7 anti-human Fas | BioLegend | 305616 |
| BV605 anti-human PD-1 | BioLegend | 367426 |
| APC anti-human TIM3 | BioLegend | 364804 |
| BV510 anti-human LAG3 | BioLegend | 369318 |
| **Immunohistochemistry** | | |
| Rabbit anti-human BCMA | Abcam | Ab199264 |
| Rabbit anti-human CS1 | Abcam | Ab237730 |
| Mouse anti-human CD138 | Dako | JY-0042 |
| Mouse anti-human CD38 | Dako | JY-0127 |
| Goat anti-mouse HPR-Ig | Abcam | Ab205720 |
| Goat anti-rabbit HPR-Ig | Abcam | Ab205718 |
| **Immunofluorescence** | | |
| Rabbit anti-human CD138 | Proteintech | 10593-1-AP |
| Rabbit anti-human BCMA | Proteintech | 27724-1-AP |
| Rabbit anti-human CS1 | Abcam | ab230945 |
| Goat anti-mouse HPR-Ig | SeraCare | 5220-0336 |

**Table S2. Prior lines of therapy for each patient**

| **Patient No.** | **Lines of prior therapies** | **Prior therapies** |
| --- | --- | --- |
| 1 | 4 | PAD (bortezomib, adriamycin, dexamethasone)  VRD (bortezomib, lenalidomide, dexamethasone)  ASCT plus maintenance with lenalidomide and bortezomib  Daratumumab, bortezomib and dexamethasone |
| 2 | 2 | VCD (bortezomib, cyclophosphamide, dexamethasone) plus maintenance with bortezomib/thalidomide/lenalidomide  PAD (bortezomib, adriamycin, daexamethasone) |
| 3 | 3 | BCD (bortezomib, cyclophosphamide, dexamethasone)  Radiation 36Gy  Ixazomib, lenalidomide and dexamethasone |
| 4 | 8 | BD (bortezomib, dexamethasone)  ASCT plus thalidomide maintenance  BCD (bortezomib, cyclophosphamide, dexamethasone) plus lenalidomide maintenance  VRD (bortezomib, lenalidomide, dexamethasone)  Ixazomib and dexamethasone  MPD (melphalan, bortezomib, dexamethasone)  PAD (bortezomib, adriamycin, dexamethasone)  Daratumumab and pomalidomide |
| 5 | 6 | TD (thalidomide, dexamethasone)  BD (bortezomib, dexamethasone)  BCD (bortezomib, cyclophosphamide, dexamethasone)  Lenalidomide and dexamethasone  Ixazomib and dexamethasone  Radiofrequency ablation |
| 6 | 2 | BCD (bortezomib, cyclophosphamide, dexamethasone)  PAD (bortezomib, Adriamycin, dexamethasone) |
| 7 | 7 | BD (bortezomib and dexamethasone)  ASCT  VRD (bortezomib, lenalidomide, dexamethasone)  VCD (lenalidomide, cyclophosphamide, dexamethasone)  Amputation of right upper limb  Anti-BCMA&CD38 bispecific CAR-T cell therapy  VDT-PACE (bortezomib, thalidomide, cisplatin, Adriamycin, etoposide) plus lenalidomide maintenance |
| 8 | 7 | PAD (bortezomib, adriamycin, dexamethasone)  BD (bortezomib and dexamethasone)  RD (lenalidomide and dexamethasone)  ICD (ixazomib, cyclophosphamide, dexamethasone)  IMP (Ixazomib, melphalan, bortezomib)  Pomalidomide plus dexamethasone  Daratumumab/3DECP (cisplatin, etoposide, ifosfamide, dexamethasone) |
| 9 | 3 | BCD (bortezomib, cyclophosphamide, dexamethasone)  ASCT plus maintenance with bortezomib and dexamethasone  Dara, lenalidomide and dexamethasone |
| 10 | 4 | VRD (bortezomib, lenalidomide, dexamethasone)  First ASCT plus maintenance with bortezomib and dexamethasone  Second ASCT plus maintenance with ixazomib and dexamethasone  Pomalidomide and bendamustine plus pomalidomide maintenance |
| 11 | 2 | BCD (bortezomib, cyclophosphamide, dexamethasone) plus lenalidomide  ASCT |
| 12 | 4 | PAD (bortezomib, Adriamycin, dexamethasone)  VRD (bortezomib, lenalidomide, dexamethasone)  DT-PACE (cytarabine, topotecan, paclitaxel) plus bortezomib  DVD (liposome doxorubicin, vincristine, dexamethasone) |
| 13 | 5 | PAD (bortezomib, adriamycin, dexamethasone)  ASCT plus thalidomide maintenance  Radiation  IRD (ixazomib, lenalidomide, dexamethasone)  PD (bortezomib and dexamethasone) |
| 14 | 4 | VDT (bortezomib, thalidomide, dexamethasone)  VRD (bortezomib, lenalidomide, dexamethasone)  BD (bortezomib, dexamethasone)  PAD (bortezomib, adriamycin, dexamethasone) |
| 15 | 7 | PAD (bortezomib, adriamycin, dexamethasone)  BD (bortezomib, dexamethasone) plus thalidomide/ lenalidomide maintenance  DVD (daratumumab, bortezomib, dexamethasone)  D-PACE (liposome doxorubicin, cisplatin, cyclophosphamide, etoposide, dexamethasone) plus lenalidomide  VRD (bortezomib, lenalidomide, dexamethasone)  KPD (carfilzomib, pomalidomide, dexamethasone)  Fully human anti-BCMA CAR-T cell therapy plus pomalidomide maintenance |
| 16 | 2 | Excision of intraspinal tumor  VD (bortezomib, dexamethasone) plus lenalidomide maintenance |
| ASCT, autologous stem cell transplantation; CAR, chimeric antigen receptor. | | |

| **Table S3. Analysis of possible relevant factors of CRS** | | | |
| --- | --- | --- | --- |
| **Possible Factors** | **CRS grade** | | |
|  | **0** | **1-3** | ***P*** |
| **Tumor burden at baseline** |  |  |  |
| β microglobulin (mg/L) | 2.9 | 5.43 | **0.0199** |
| Serum albumin (g/L) | 36.09 | 31.48 | 0.0759 |
| Lactic dehydrogenase (U/L) | 195.1 | 694.7 | 0.2025 |
| Serum M protein (g/L) | 10.94 | 16.05 | 0.6142 |
| MM cells in BM by morphology (%) | 13 | 29.5 | 0.1981 |
| MM cells in BM by immunophenotyping (%) | 6.43 | 21.92 | 0.1214 |
| BCMA+ ratio in MM cells (%) | 86.94 | 65.2 | **0.0338** |
| CS1+ ratio in MM cells (%) | 98.01 | 96.42 | 0.2328 |
| sBCMA in PB (pg/mL) | 2492 | 3737 | 0.6089 |
| sBCMA in BM (pg/mL) | 737.4 | 3724 | 0.2339 |
| R-ISS stage |  |  | 0.5879 |
| I-II | 4 | 1 |  |
| III | 6 | 5 |  |
| **Prior treatment** |  |  |  |
| Lines of prior treatment | 3.6 | 5.5 | 0.0716 |
| Prior ASCT |  |  | 0.6329 |
| Yes | 5 | 2 |  |
| No | 5 | 4 |  |
| Prior CAR-T cell therapy |  |  | >0.9999 |
| Yes | 1 | 1 |  |
| No | 9 | 5 |  |
| Prior Daratumumab |  |  | 0.6066 |
| Yes | 3 | 3 |  |
| No | 7 | 3 |  |
| **Cytokines and inflammatory markers** |  |  |  |
| Baseline serum IL-2 level (pg/mL) | 2.00 | 1.84 | 0.7676 |
| Peak serum IL-2 level (pg/mL) | 2.86 | 22.98 | 0.1764 |
| Ratio of peak to baseline of serum IL-2 | 1.67 | 14.17 | 0.1185 |
| Baseline serum IL-4 level (pg/mL) | 1.93 | 1.99 | 0.9185 |
| Peak serum IL-4 level (pg/mL) | 2.81 | 2.57 | 0.6278 |
| Ratio of peak to baseline of serum IL-4 | 2.23 | 1.55 | 0.4685 |
| Baseline serum IL-6 level (pg/mL) | 9.44 | 11.79 | 0.6084 |
| Peak serum IL-6 level (pg/mL) | 43.27 | 1262 | 0.0586 |
| Ratio of peak to baseline of serum IL-6 | 9.27 | 147.1 | **0.0439** |
| Baseline serum IL-10 level (pg/mL) | 4.37 | 6.44 | 0.2446 |
| Peak serum IL-10 level (pg/mL) | 66.45 | 102.7 | 0.3981 |
| Ratio of peak to baseline of serum IL-10 | 21.15 | 21.43 | 0.9798 |
| Baseline serum TNF-α level (pg/mL) | 1.35 | 1.54 | 0.6154 |
| Peak serum TNF-α level (pg/mL) | 2.11 | 34.28 | 0.2059 |
| Ratio of peak to baseline of serum TNF-α | 2.28 | 15.60 | 0.2246 |
| Baseline serum IFN-γ level (pg/mL) | 1.64 | 1.61 | 0.9458 |
| Peak serum IFN-γ level (pg/mL) | 3.27 | 7.85 | **0.0047** |
| Ratio of peak to baseline of serum IFN-γ | 2.55 | 6.28 | **0.0218** |
| Baseline serum ferritin level (ng/mL) | 551.3 | 2832 | 0.0839 |
| Peak serum ferritin level (ng/mL) | 2580 | 16552 | **0.0100** |
| Ratio of peak to baseline of serum ferritin | 3.94 | 10.23 | **0.0046** |
| Baseline serum CRP level (mg/L) | 9.68 | 15.36 | 0.3529 |
| Peak serum CRP level (mg/L) | 39.27 | 60.56 | 0.3578 |
| Ratio of peak to baseline of serum CRP | 6.99 | 24.28 | 0.2899 |
| Descriptive statistics include means for continuous variables and counts for categorical variables. Unpaired t test was used for continuous variables, and Fisher’s exact test was used for categorical variables. CRS: cytokine release syndrome; MM: multiple myeloma; BM: bone marrow; PB: peripheral blood; ASCT: autologous stem cell transplantation; CAR: chimeric antigen receptor; IL: interleukin; TNF: tumor necrosis factor; IFN: interferon; CRP: C reaction protein. | | | |

| **Table S4. Analysis of possible relevant factors of remission depth** | | | |
| --- | --- | --- | --- |
| **Possible Factors** | **Clinical Response** | | |
|  | **sCR** | **VGPR/PR** | ***P*** |
| Tumor burden at baseline |  |  |  |
| β microglobulin (mg/L) | 2.65 | 5.07 | 0.0563 |
| Serum albumin (g/L) | 34.80 | 32.83 | 0.5365 |
| Lactic dehydrogenase (U/L) | 183.20 | 614.70 | 0.3697 |
| Serum M protein (g/L) | 14.72 | 16.09 | 0.9080 |
| MM cells in BM by morphology (%) | 14.50 | 31.43 | 0.2389 |
| MM cells in BM by immunophenotyping (%) | 8.16 | 20.98 | 0.2760 |
| BCMA+ ratio in MM cells (%) | 80.41 | 73.91 | 0.5644 |
| CS1+ ratio in MM cells (%) | 97.94 | 96.71 | 0.3623 |
| sBCMA in PB (pg/mL) | 1114.00 | 3109.00 | 0.3682 |
| sBCMA in BM (pg/mL) | 2019.00 | 2199.00 | 0.9445 |
| R-ISS stage |  |  | >0.9999 |
| I-II | 1 | 2 |  |
| III | 5 | 5 |  |
| Prior treatment |  |  |  |
| Lines of prior treatment | 3.50 | 4.43 | 0.4177 |
| Prior ASCT |  |  | 0.5921 |
| Yes | 3 | 2 |  |
| No | 3 | 5 |  |
| Prior CAR-T cell therapy |  |  | >0.9999 |
| Yes | 0 | 1 |  |
| No | 6 | 6 |  |
| Prior Daratumumab |  |  | 0.5594 |
| Yes | 1 | 3 |  |
| No | 5 | 4 |  |
| Cytokines and inflammatory markers |  |  |  |
| Baseline serum IL-2 level (pg/mL) | 1.76 | 2.13 | 0.5726 |
| Peak serum IL-2 level (pg/mL) | 2.59 | 20.44 | 0.3268 |
| Ratio of peak to baseline of serum IL-2 | 2.01 | 12.20 | 0.3005 |
| Baseline serum IL-4 level (pg/mL) | 1.73 | 2.14 | 0.5800 |
| Peak serum IL-4 level (pg/mL) | 2.91 | 2.76 | 0.8019 |
| Ratio of peak to baseline of serum IL-4 | 3.11 | 1.36 | 0.0985 |
| Baseline serum IL-6 level (pg/mL) | 4.63 | 14.89 | **0.0302** |
| Peak serum IL-6 level (pg/mL) | 43.98 | 1081.00 | 0.1918 |
| Ratio of peak to baseline of serum IL-6 | 11.17 | 124.70 | 0.1804 |
| Baseline serum IL-10 level (pg/mL) | 2.45 | 6.56 | **0.0138** |
| Peak serum IL-10 level (pg/mL) | 54.42 | 125.80 | 0.1298 |
| Ratio of peak to baseline of serum IL-10 | 25.33 | 24.91 | 0.9730 |
| Baseline serum TNF-α level (pg/mL) | 1.25 | 1.56 | 0.5136 |
| Peak serum TNF-α level (pg/mL) | 2.10 | 29.71 | 0.3755 |
| Ratio of peak to baseline of serum TNF-α | 2.89 | 13.54 | 0.4281 |
| Baseline serum IFN-γ level (pg/mL) | 1.44 | 1.69 | 0.6272 |
| Peak serum IFN-γ level (pg/mL) | 3.25 | 7.19 | 0.0501 |
| Ratio of peak to baseline of serum IFN-γ | 3.12 | 5.52 | 0.2286 |
| Baseline serum ferritin level (ng/mL) | 1154.00 | 2006.00 | 0.6081 |
| Peak serum ferritin level (ng/mL) | 7248.00 | 11208.00 | 0.5749 |
| Ratio of peak to baseline of serum ferritin | 5.22 | 9.11 | 0.1389 |
| Baseline serum CRP level (mg/L) | 8.92 | 12.41 | 0.5624 |
| Peak serum CRP level (mg/L) | 46.89 | 40.51 | 0.7824 |
| Ratio of peak to baseline of serum CRP | 8.97 | 20.72 | 0.5568 |
| Descriptive statistics include means for continuous variables and counts for categorical variables. Unpaired t test was used for continuous variables, and Fisher’s exact test was used for categorical variables. sCR, stringent complete response; VGPR: very good partial response; PR: partial response; MM: multiple myeloma; BM: bone marrow; PB: peripheral blood; ASCT: autologous stem cell transplantation; CAR: chimeric antigen receptor; IL: interleukin; TNF: tumor necrosis factor; IFN: interferon; CRP: C reaction protein. | | | |

| **Table S5. Information of infused CS1-BCMA CAR-T cells** | | | | | |
| --- | --- | --- | --- | --- | --- |
| **ID** | **Infused dose (CAR+T cells/kg)** | **CAR+/CD3+ T cells** | **CD4+/CAR+CD3+ T cells** | **CD8+/CAR+CD3+ T cells** | **Total number of infused CAR-T cells** |
| 1 | 0.75×106 | 53.00% | 61.60% | 35.60% | 0.38×108 |
| 2 | 0.75×106 | 64.00% | 67.50% | 28.20% | 0.51×108 |
| 3 | 0.75×106 | 59.90% | 43.30% | 54.00% | 0.54×108 |
| 4 | 1.50×106 | 42.00% | 45.70% | 47.10% | 1.00×108 |
| 5* | 1.50×106 | 58.10% | 64.90% | 33.30% | 0.83×108 |
| 6 | 1.50×106 | 78.00% | 69.70% | 28.70% | 1.01×108 |
| 7 | 1.50×106 | 34.90% | 30.90% | 65.30% | 0.77×108 |
| 8 | 1.50×106 | 75.80% | 88.50% | 10.50% | 0.74×108 |
| 9 | 1.50×106 | 49.00% | 69.10% | 28.60% | 0.71×108 |
| 10 | 3.0×106 | 41.00% | 23.30% | 71.10% | 2.61×108 |
| 11 | 3.0×106 | 61.30% | 73.70% | 22.80% | 1.35×108 |
| 12 | 3.0×106 | 48.70% | 56.10% | 40.00% | 1.65×108 |
| 13 | 3.0×106 | 40.00% | 35.10% | 57.60% | 1.41×108 |
| 14 | 3.0×106 | 57.70% | 59.70% | 35.40% | 1.92×108 |
| 15 | 3.0×106 | 43.10% | 70.70% | 28.40% | 2.25×108 |
| 16 | 3.0×106 | 77.80% | 75.40% | 22.20% | 1.74×108 |
| mean | 2.0×106 | 55.27% | 58.45% | 38.05% | 1.21×108 |
| *Patient 5 had grade 3 cytokine release syndrome and grade 3 gastrointestinal bleeding, which was judged as the dose limiting toxicity. Thus, another three patients were included at the dose of 1.50×106 CAR+T cells/kg. | | | | | |

| **Table S6. Phenotypic characteristics of infused CAR-T cells** | | | |
| --- | --- | --- | --- |
| Proportion (%) | CAR^+^CD3^+^T | CAR^-^CD3^+^T | *P* |
| CD4^+^ | 43.47 (34.41-52.52) | 22.44 (15.96-28.93) | **<0.0001** |
| CD8^+^ | 44.18 (35.18-53.17) | 67.47 (59.36-75.58) | **<0.0001** |
| 45RA^+^62L^+^ | 32.20 (22.60-41.80) | 34.82 (19.76-49.87) | 0.5691 |
| 45RA^-^62L^+^ | 5.01 (2.46-7.57) | 5.63 (2.85-8.42) | 0.4707 |
| 45RA^-^62L^-^ | 9.43 (7.53-11.32) | 32.89 (13.30-52.49) | **0.0202** |
| 45RA^+^62L^-^ | 53.36 (42.50-64.22) | 26.65 (17.44-35.86) | **0.0013** |
| CS1^+^ | 64.85 (53.19-76.52) | 64.62 (51.90-77.35) | 0.8661 |
| CS1^+^CD8^+^ | 70.99 (58.04-83.94) | 67.90 (54.76-81.04) | 0.0512 |
| CCR3^+^CD4^+^ | 49.95 (33.40-66.50) | 31.90 (20.41-43.38) | **0.0004** |
| CXCR4^+^CD4^+^ | 42.20 (30.81-53.99) | 40.45 (30.54-50.35) | 0.7008 |
| Regulatory T cells | 12.9 (8.09-17.71) | 0.57 (0.06-1.079) | **<0.0001** |
| Fas^+^ | 98.13 (96.72-99.53) | 97.71 (96.43-98.99) | 0.3629 |
| LAG3^+^ | 7.24 (1.92-12.57) | 6.78 (1.12-12.45) | 0.3870 |
| PD1^+^ | 3.37 (1.47-5.26) | 1.57 (0.87-2.26) | **0.0354** |
| TIM3^+^ | 61.96 (56.47-67.45) | 54.20 (46.41-61.99) | **0.0078** |

Descriptive statistics include means and 90% confidence interval. Paired t test was used.

| **Table S7. Subgroup analysis of CAR-T phenotypes and clinical response** | | | | | |
| --- | --- | --- | --- | --- | --- |
| Phenotypes | Proportion (%) | sCR | VGPR/PR | PD | *P* |
| CAR^+^CD3^+^T | CAR^+^ | 46.52 | 45.03 | 34.12 | 0.4436 |
|  | CAR^+^CD4^+^ | 41.49 | 47.57 | 37.85 | **0.0212** |
|  | CAR^+^CD8^+^ | 43.81 | 42.42 | 49.01 | 0.8608 |
|  | 45RA^+^62L^+^ | 39.28 | 24.68 | 35.57 | 0.3473 |
|  | 45RA^-^62L^+^ | 4.89 | 4.76 | 5.84 | 0.9520 |
|  | 45RA^-^62L^-^ | 8.72 | 10.34 | 8.70 | 0.6925 |
|  | 45RA^+^62L^-^ | 47.10 | 60.20 | 49.90 | 0.5187 |
|  | CS1^+^ | 64.31 | 61.61 | 73.50 | 0.7585 |
|  | CS1^+^CD8^+^ | 66.78 | 71.94 | 77.17 | 0.8447 |
|  | CCR3^+^CD4^+^ | 55.61 | 56.20 | 24.02 | 0.2941 |
|  | CXCR4^+^CD4^+^ | 50.87 | 35.81 | 40.83 | 0.4881 |
|  | Regulatory T cells | 14.65 | 11.55 | 12.56 | 0.8435 |
|  | Fas^+^ | 98.43 | 98.50 | 96.63 | 0.5858 |
|  | LAG3^+^ | 7.40 | 4.39 | 13.56 | 0.4420 |
|  | PD1^+^ | 3.54 | 2.56 | 4.90 | 0.6570 |
|  | TIM3^+^ | 68.45 | 61.93 | 49.03 | **0.0154** |
| CAR-CD3+T | CAR^-^ | 53.17 | 54.80 | 64.78 | 0.4894 |
|  | CAR^-^CD4^+^ | 19.39 | 28.37 | 14.73 | 0.1404 |
|  | CAR^-^CD8^+^ | 68.77 | 61.27 | 79.33 | 0.2042 |
|  | 45RA^+^62L^+^ | 39.70 | 23.01 | 52.60 | 0.2914 |
|  | 45RA^-^62L^+^ | 6.18 | 4.94 | 6.16 | 0.9082 |
|  | 45RA^-^62L^-^ | 26.26 | 49.85 | 6.60 | 0.2084 |
|  | 45RA^+^62L^-^ | 27.82 | 22.22 | 34.67 | 0.6003 |
|  | CS1^+^ | 61.28 | 62.16 | 77.07 | 0.6366 |
|  | CS1^+^CD8^+^ | 62.10 | 67.93 | 79.43 | 0.6422 |
|  | CCR3^+^CD4^+^ | 38.49 | 32.89 | 16.40 | 0.3702 |
|  | CXCR4^+^CD4^+^ | 41.93 | 35.10 | 49.93 | 0.5294 |
|  | Regulatory T cells | 0.56 | 0.83 | 0.00 | 0.4835 |
|  | Fas^+^ | 97.23 | 98.56 | 96.70 | 0.4711 |
|  | LAG3^+^ | 6.53 | 4.56 | 12.47 | 0.5900 |
|  | PD1^+^ | 0.89 | 1.66 | 2.71 | 0.1391 |
|  | TIM3^+^ | 62.67 | 50.10 | 46.83 | 0.1960 |

Descriptive statistics include means. One-Way ANOVA was used.

| **Table S8. Subgroup analysis of CAR-T phenotypes and CRS occurrence** | | | | |
| --- | --- | --- | --- | --- |
| Phenotypes | CRS | No | Yes | *P* |
| CAR^+^CD3^+^T | CAR^+^ | 42.90 | 44.61 | 0.8247 |
|  | CAR^+^CD4^+^ | 42.69 | 44.76 | 0.8227 |
|  | CAR^+^CD8^+^ | 43.06 | 46.04 | 0.7450 |
|  | 45RA^+^62L^+^ | 32.84 | 31.12 | 0.8606 |
|  | 45RA^-^62L^+^ | 5.45 | 4.28 | 0.6530 |
|  | 45RA^-^62L^-^ | 10.55 | 7.56 | 0.1048 |
|  | 45RA^+^62L^-^ | 51.16 | 57.02 | 0.5955 |
|  | CS1^+^ | 67.8 | 59.95 | 0.5067 |
|  | CS1^+^CD8^+^ | 71.78 | 69.67 | 0.873 |
|  | CCR3^+^CD4^+^ | 47.93 | 53.32 | 0.7492 |
|  | CXCR4^+^CD4^+^ | 44.99 | 38.08 | 0.5569 |
|  | Regulatory T cells | 12.88 | 12.94 | 0.9910 |
|  | Fas^+^ | 98.12 | 98.13 | 0.9926 |
|  | LAG3^+^ | 8.75 | 4.73 | 0.4551 |
|  | PD1^+^ | 3.72 | 2.78 | 0.6267 |
|  | TIM3^+^ | 58.80 | 67.22 | 0.1162 |
| CAR-CD3+T | CAR^-^ | 56.56 | 55.22 | 0.8566 |
|  | CAR^-^CD4^+^ | 19.20 | 27.86 | 0.1343 |
|  | CAR^-^CD8^+^ | 70.30 | 62.74 | 0.3358 |
|  | 45RA^+^62L^+^ | 39.14 | 27.61 | 0.4483 |
|  | 45RA^-^62L^+^ | 24.99 | 46.07 | 0.2817 |
|  | 45RA^-^62L^-^ | 4.96 | 6.75 | 0.5259 |
|  | 45RA^+^62L^-^ | 30.90 | 19.58 | 0.2155 |
|  | CS1^+^ | 68.54 | 58.10 | 0.4160 |
|  | CS1^+^CD8^+^ | 69.55 | 65.15 | 0.7425 |
|  | CCR3^+^CD4^+^ | 31.65 | 32.30 | 0.9559 |
|  | CXCR4^+^CD4^+^ | 40.33 | 40.63 | 0.9762 |
|  | Regulatory T cells | 0.09 | 1.38 | **0.0038** |
|  | Fas^+^ | 97.96 | 97.30 | 0.6116 |
|  | LAG3^+^ | 7.71 | 5.23 | 0.6669 |
|  | PD1^+^ | 1.85 | 1.10 | 0.2815 |
|  | TIM3^+^ | 52.12 | 57.67 | 0.4815 |
| Descriptive statistics include means. Paired t test was used. | | | | |

| **Patient No.** | **Clinical outcomes** | **PB** | | | **BM** | | |
| --- | --- | --- | --- | --- | --- | --- | --- |
|  |  | D0 | D14-M3 | M9-M12/PD | D0 | D14-M3 | M9-M12/PD |
| **2** | sCR+ | <0.312 | <0.312 | <0.312 | <0.312 | <0.312 | **8.17** |
| **3** | sCR+ | <0.312 | <0.312 | <0.312 | <0.312 | <0.312 | <0.312 |
| **4** | sCR, lost in M6 | <0.312 | <0.312 | **/** | <0.312 | <0.312 | **/** |
| **5** | VGPR, lost in M2 | <0.312 | >2000 | **/** | <0.312 | >2000 | **/** |
| **6** | sCR+ | <0.312 | <0.312 | <0.312 | <0.312 | <0.312 | <0.312 |
| **7** | PD, withdrawal in M1 | <0.312 | <0.312 | **/** | **/** | **/** | **/** |
| **8** | PD, withdrawal in M2 | <0.312 | <0.312 | **/** | **/** | **/** | **/** |
| **9** | PD, withdrawal in M2 | <0.312 | <0.312 | **/** | **/** | **/** | **/** |
| **10** | sCR, relapse in M9 | <0.312 | <0.312 | <0.312 | **50.63** | <0.312 | **1.47** |
| **11** | sCR+ | <0.312 | <0.312 | <0.312 | <0.312 | <0.312 | <0.312 |
| **12** | PR, PD in M2 | **4.83** | <0.312 | **/** | <0.312 | <0.312 | **/** |
| **13** | PR, lost in M6 | <0.312 | <0.312 | **/** | <0.312 | **390.10** | **/** |
| **14** | PR, lost in M6 | <0.312 | <0.312 | **/** | <0.312 | **36.80** | **/** |
| **15** | VGPR, PD in M6 | <0.312 | <0.312 | <0.312 | <0.312 | <0.312 | <0.312 |

**Table S9. Longitudinal changes of sBCMA in the PB and BM after CS1-BCMA CAR-T cell infusion**

PB: peripheral blood; BM: bone marrow; D: day; M: month; sCR: stringent complete response; VGPR: very good partial response; PD: progressive disease.

**Supplemental Figures
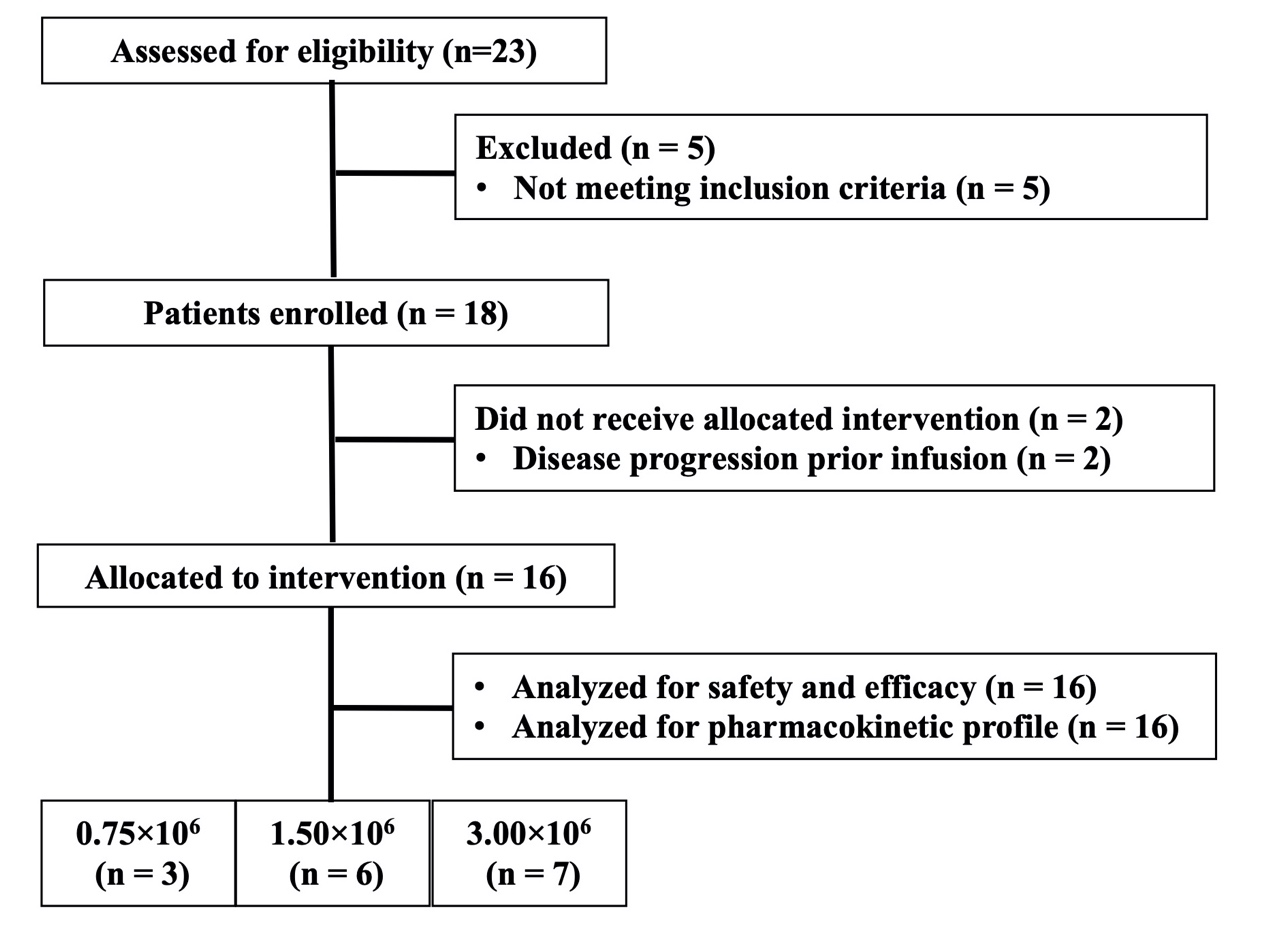
**

**Fig. S1 Consort diagram.**

**
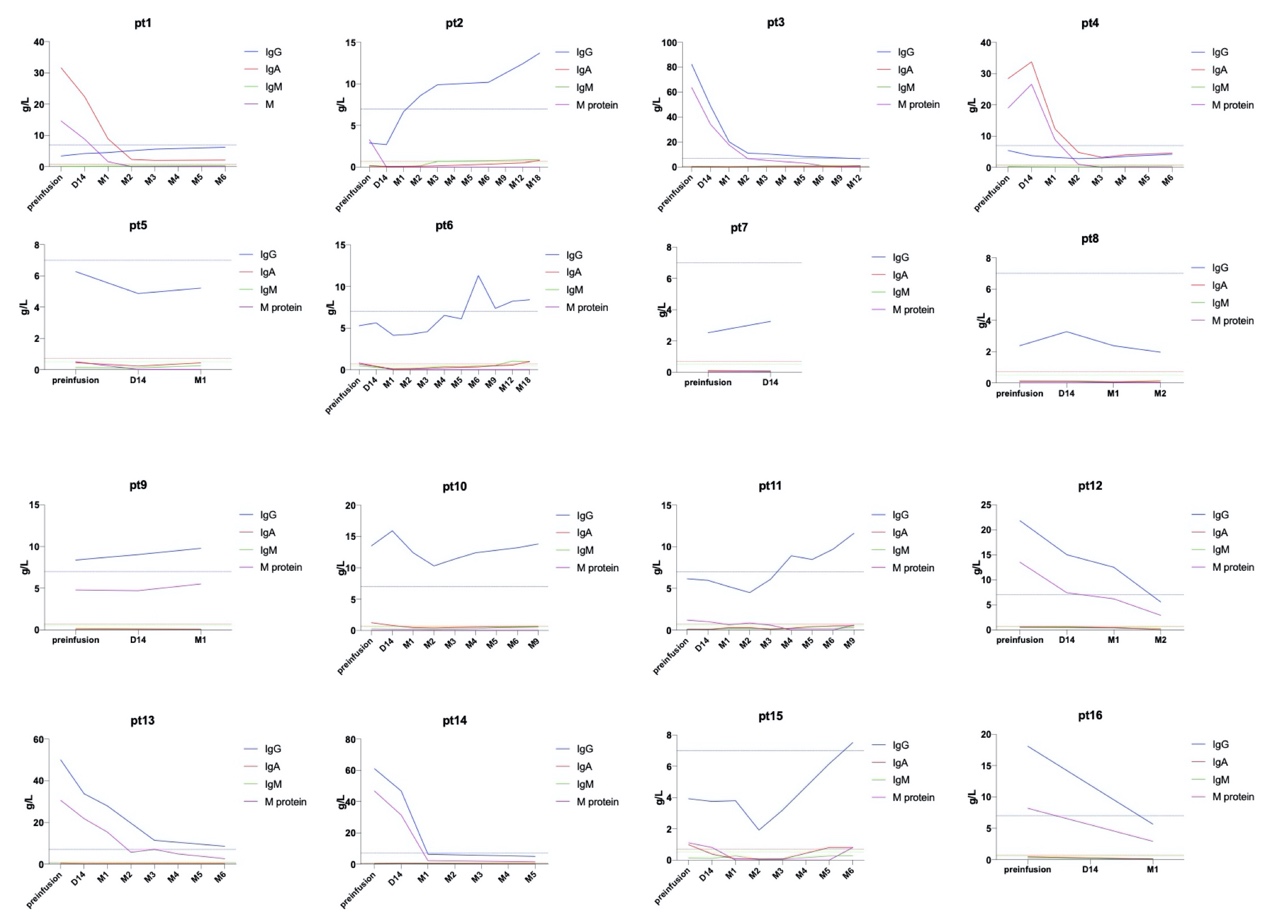
Fig. S2 Immunoglobulin levels following CS1-BCMA CAR-T cell infusion.** Normal range of serum immunoglobulin (Ig) is as follows: IgG 7.0–16.0 g/L; IgA 0.7–4.0 g/L; IgM 0.5–2.2 g/L. M protein: monoclonal immunoglobulin; pt: patient; D: day; M: month. Dashed lines represent the lower normal limit of the corresponding Ig.

**Fig. S3 Correlation analysis of in vivo kinetics of CS1-BCMA CAR-T cells.** **A** Pearson correlation analysis of CAR copies/ug DNA in peripheral blood (PB) and bone marrow (BM) by digital droplet PCR. **B** Pearson correlation analysis of CAR+T cells/uL in PB and BM by flow cytometry. **C** Pearson correlation analysis of CAR copies/ug DNA and CAR+T cells/uL in PB. **D** Pearson correlation analysis of CAR copies/ug DNA and CAR+T cells/uL in BM.

**
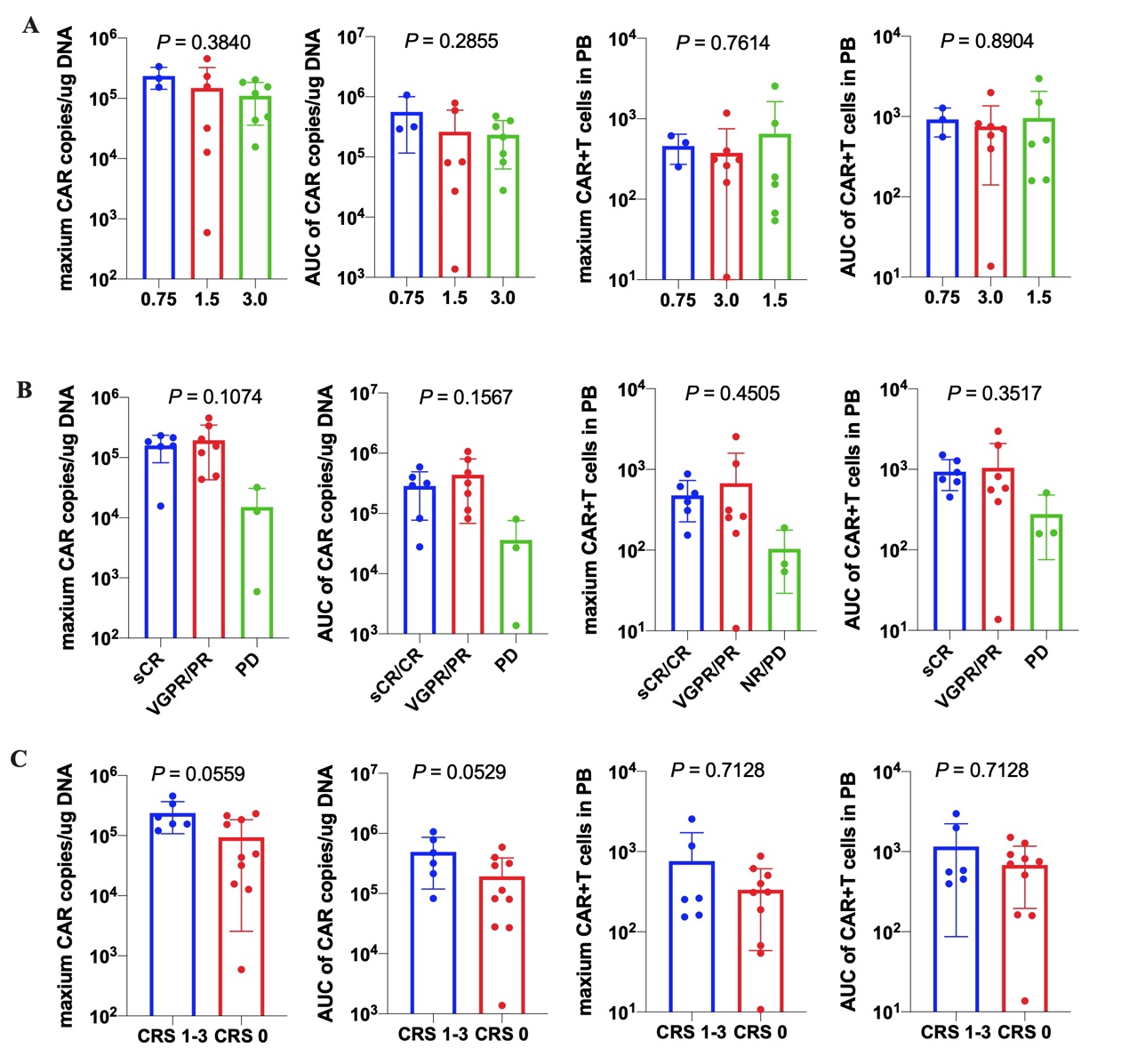
**

**Fig.S4 Correlation analysis of in vivo expansion of CS1-BCMA CAR-T cells with clinical efficacy and cytokines release syndrome (CRS). A** Subgroup analysis of peak expansion and the expansion curve in the first 28 days after infusion (AUC) with the infused dose of CAR-T cells. One-way ANOVA was used. **B** Subgroup analysis of peak expansion and AUC with clinical remission depth. sCR: stringent complete response; VGPR: very good partial response; PR: partial response; PD: progressive disease. One-way ANOVA was used. **C** Subgroup analysis of peak expansion and AUC with CRS occurrence. Unpaired t test was used.
